# Silencing *Itch* in human peripheral blood monocytes promotes their differentiation into osteoclasts

**DOI:** 10.1007/s11033-022-07726-1

**Published:** 2022-07-06

**Authors:** O. J. Read, D. J. Harrison

**Affiliations:** Pathology Department, School of Medicine, University of St Andrews, St Andrews, UK

**Keywords:** Itch, Monocyte, Osteoclast, E3 ubiquitin ligase, Knockdown

## Abstract

**Introduction:**

Two clinical case reports of humans with mutations in *Itch* reported distinct morphological defects such as stunted growth, macrocephaly, and dysmorphic features indicating a role for *Itch* in bone remodelling. Studies in mice have found that the encoded E3 ubiquitin ligase acts as a negative regulator of osteoclastogenesis, however no studies have investigated whether this is translatable to a human model.

**Experimental procedures:**

Human peripheral blood monocytes were separated from whole blood and grown in M-CSF containing media. Media was later supplemented with RANKL to promote osteoclast differentiation. Transient siRNA-mediated *Itch* knockdown (si-Itch) in monocytes was verified by qPCR and western blot to confirm reduction in both *Itch* mRNA and protein respectively. Monocytes were aliquoted onto 96-well plates where confluence and osteoclast formation were analysed using automated cytometry analysis before and after staining for tartrate resistant acid phosphatase activity (TRAP). Cells were also stained with Hoechst33342 to look for multinucleate cells.

**Results:**

Cells treated with si-Itch showed an 80% knockdown in *Itch* mRNA and > 75% reduction in protein. Following the 7-day differentiation period, si-Itch caused a 47% increase in multinucleate cells and a 17% increase in numbers of large cellular bodies and, indicating an overall increase in mature osteoclast formation.

**Conclusions:**

Our preliminary data shows silencing *Itch* expression increases the potential of primary human monocytes to differentiate into osteoclast-like cells in vitro.

**Supplementary Information:**

The online version contains supplementary material available at 10.1007/s11033-022-07726-1.

## Introduction

The *Itch* gene encodes a Hect domain E3 ubiquitin ligase that was originally identified by Perry’s group in their study of non-agouti lethal mice [1]. These *Itch*-knockout (*Itch*-/-) mice were dubbed “Itchy” mice due to the excessive scratching phenotype they displayed along with other more severe autoimmune defects [1, 2]. Since its discovery *Itch* has been shown to have several roles in regulating autoimmunity including the T-cell antigen receptor response, T-cell anergy, and differentiation of T-helper type 2 cells (T_H_2), T-follicular helper cells (T_FH_), and regulatory T-cells (T-reg) [2–4].

Clinical significance of *Itch* in humans was not apparent until a case study where Lohr et al. followed a small closed group of children of old Amish heritage who presented with numerous symptoms including organomegaly, failure to thrive, stunted growth, macrocephaly, dysmorphic features, and inflammatory cell infiltration of lungs liver and gut [5]. Genome-wide autozygosity mapping and subsequent sequence analysis identified a single nucleotide insertion in exon 6 of the *Itch* gene, resulting in a frameshift mutation that causes the translated protein to lack three of the four WW binding motifs in addition to the catalytically active HECT domain [5]. A different report described an individual with biallelic mutations in *Itch* resulting in a truncated version of the E3 ligase, who displayed the same defects with the addition of camptodactyly of the fingers [6]. Whilst Itch’s role in regulating immunity is well documented, the other associated features i.e., stunted growth, dysmorphic features, etc., indicates a role for *Itch* in bone growth and remodelling.

Mice have been used for the study of human skeletal disease as there are many similarities between the mouse and human skeletal system including the mechanisms and genes involved in bone development and metabolism [7, 8]. The accelerated bone growth and remodelling rate in mice due to their considerably shorter lifespan also makes them a convenient model. Studies comparing *Itch*-/- mice to wild-type (WT) have observed that *Itch-/-* mice have greater average numbers of both osteoblasts and osteoclasts [9–11], cells that govern bone formation and bone resorption respectively. Dysregulation of these opposing processes results in osteopetrosis or osteolysis which are associated with debilitating diseases. Whether or not *Itch-/-* mice were osteopetrotic or osteoporotic seemed age-dependent: young mice have greater bone density whilst older mice have lower bone density [9, 11].

The discovery of receptor activator of nuclear factor-κB (RANK) and osteoprotegrin (OPG) and their role in osteoclastogenesis was a breakthrough in understanding the bone remodelling process [12, 13]. Interaction of RANK with RANKL (RANK ligand), secreted by osteoblasts and stromal cells in-vivo [14], promotes osteoclast differentiation by inducing autoubiquitination of TNF receptor-associated factor 6 (TRAF6), upregulating nuclear factor-κB (NF-κB) transcriptional activity [9, 15, 16]. *TRAF6-/-* mice have impaired osteoclast function resulting in osteopetrosis [17], causing defects in bone remodelling and tooth eruption. Studies in mice show that *Itch* negatively regulates osteoclast differentiation by associating with the deubiquitinating (DUB) enzyme cylindromatosis (CYLD). The Itch/CYLD complex removes Ub from TRAF6 to prevent further NF-κB signalling. CYLD-/- mice are hypersensitive to RANKL, have prolonged NF-κB signalling, and severe osteoporosis [18].

A recent study showed that Itch is negatively regulated in osteoarthritic (OA) tissue and that overexpression of *Itch* in human chondrocytes stimulated with LPS resulted in reduced apoptosis and extracellular matrix degradation by inhibiting *Notch* signalling [19]. Overexpression of Itch in OA mouse chondrocytes alleviated OA progression showing the parallels between human and mouse settings [19]. However, the translation of *Itch* regulation of osteoclast formation in humans from mice has not been explored.

We hypothesise that genetic knockdown of *Itch* in human peripheral blood monocytes facilitates differentiation into mature osteoclasts. Monocytes isolated from whole blood were cultured and subject to transient *Itch* knockdown using siRNA before being exposed to culture conditions to promote osteoclast differentiation. Different staining methods and automated cytometry analysis were then used to assess osteoclast formation.

## Materials and methods

### Monocyte isolation from whole blood

Whole blood was collected from healthy volunteers using a protocol approved by School of Medicine’s research ethics committee (MD9202). 10% EDTA was added to blood (10 μl per ml of blood) to prevent coagulation. Blood was diluted 1:1 with PBS, layered on top of 10 ml Histopaque (Sigma Aldrich), transferred to a centrifuge, and spun at 800×*g* for 30 min (no brake). The buffy coat layer was aspirated and washed 2–3 times with PBS, centrifuging at 300×*g* for 10 min (brake applied), discarding supernatant each time. Residual monocytes were resuspended in culture media at a density of 10^6^ cells/ml and dispensed onto either 6-well plates (Corning) at 10^6^ cells/well, plain-surface or Osteo Assay Surface 96-well plates (Corning) at 1000 cells/well.

### Cell culture

Monocytes were cultured in α-MEM (ThermoFisher) culture media supplemented with 10% FBS, 1% Penicillin/Streptomycin and 20 ng/ml macrophage colony-stimulating factor (M-CSF—R&D Biosciences). Cells were incubated with 5% CO_2_ at 37 °C and media was changed every 3 days. To promote osteoclast differentiation the media was further supplemented with 50 ng/ml RANKL (R&D Biosciences) after which media was changed every 2 days.

### siRNA transfection

*Itch*-targeting siRNA duplex data was kindly supplied by Dr Simon Newman, Nanogenics Ltd. And synthesised by Eurogentec. Sense; 5’-GCUGUUGUUUGCCAUAGAA-3’, antisense; 5’-UUCUAUGGCAAACAACAGC-3’. For a scrambled control we used a commercial negative control (Eurogentec). siRNA was combined with Lipofectamine RNAiMAX (ThermoFisher) in OPTImem to get a solution with a final concentration of 125 nM. After 6 days of PBMC culture siRNA solution was added to cells to a final concentration of 12.5 nM. After 24 h media was aspirated and replaced with fresh, RANKL-containing media. siRNA was re-applied after 72 h RANKL exposure.

### Western blot

Lysates from 6-well plates were taken 48 h after siRNA transfection using RIPA buffer. BCA assay was used to determine protein concentration for each sample to make aliquots for SDS–polyacrylamide gel electrophoresis (SDS-PAGE). Antibodies used: rabbit anti-Itch D8Q6D (CST), mouse anti-β-actin 8H10D10 (CST), donkey anti-rabbit CW800 (Licor), and donkey anti-mouse RD680 (Licor). Membranes were imaged and quantified using a Licor Odyssey Scanner (Licor).

### RNA extraction and analysis by qPCR

Lysates from 6-well plates were taken 48 h after siRNA transfection. RNA extraction and isolation, reverse transcription, and qPCR master mix preparation was performed using Qiagen’s RNEasy Mini Kit, QuantiTect Reverse Transcription Kit and Rotor-Gene SYBR Green PCR Kit respectively (Qiagen) as per the manufacturer’s instructions. Primers used for relative qPCR were QuantiTECT Primer Assays (Qiagen) for *GAPDH* (housekeeping gene) and *Itch*. qPCR was performed using a Rotor-Gene Q (Qiagen). Relative *Itch* expression was determined using the ΔΔCt method.

### Tartrate resistant acid phosphatase (TRAP) staining

Osteoclasts were stained for TRAP activity using the Acid Phosphatase Leukocyte Kit (Sigma Aldrich). Preparation and staining were done as per the manufacturer’s instructions however the protocol was adapted for 96-well plates. Cells were viewed under a light-microscope and images taken using Leica Application Suite V4 (Leica). Automated cytometry analysis (Celigo, Nexcelcom) was performed on the plates to quantify TRAP activity. Analysis algorithms were designed to screen for: A. all objects with TRAP^+^ activity and B. large multicellular objects with TRAP^+^ activity (positive findings with this algorithm were checked manually).

### Nuclear staining using Hoechst 33,342

After TRAP staining, wells were air-dried and incubated with 100 μl of Hoechst staining solution (1:10,000 Hoechst 33342—Life Technologies—in PBS) in the dark for 15 min at room temperature. Wells were imaged using automated cytometry analysis software and multinucleate cells were manually counted and recorded in a spreadsheet for further analysis.

### Statistical analysis

Graphs were generated in Graph Pad (version 9.3.1). To test for significance between the means of the test and control conditions Students’ t-test was used.

## Results

### Successful transient knockdown of *Itch* in human monocytes

Osteoclasts can be differentiated from monocytes derived from whole-blood samples, provided there are suitable growth conditions to encourage monocyte isolation [14]. Monocytes were isolated and cultured with media containing M-CSF.

**Fig. 1:**
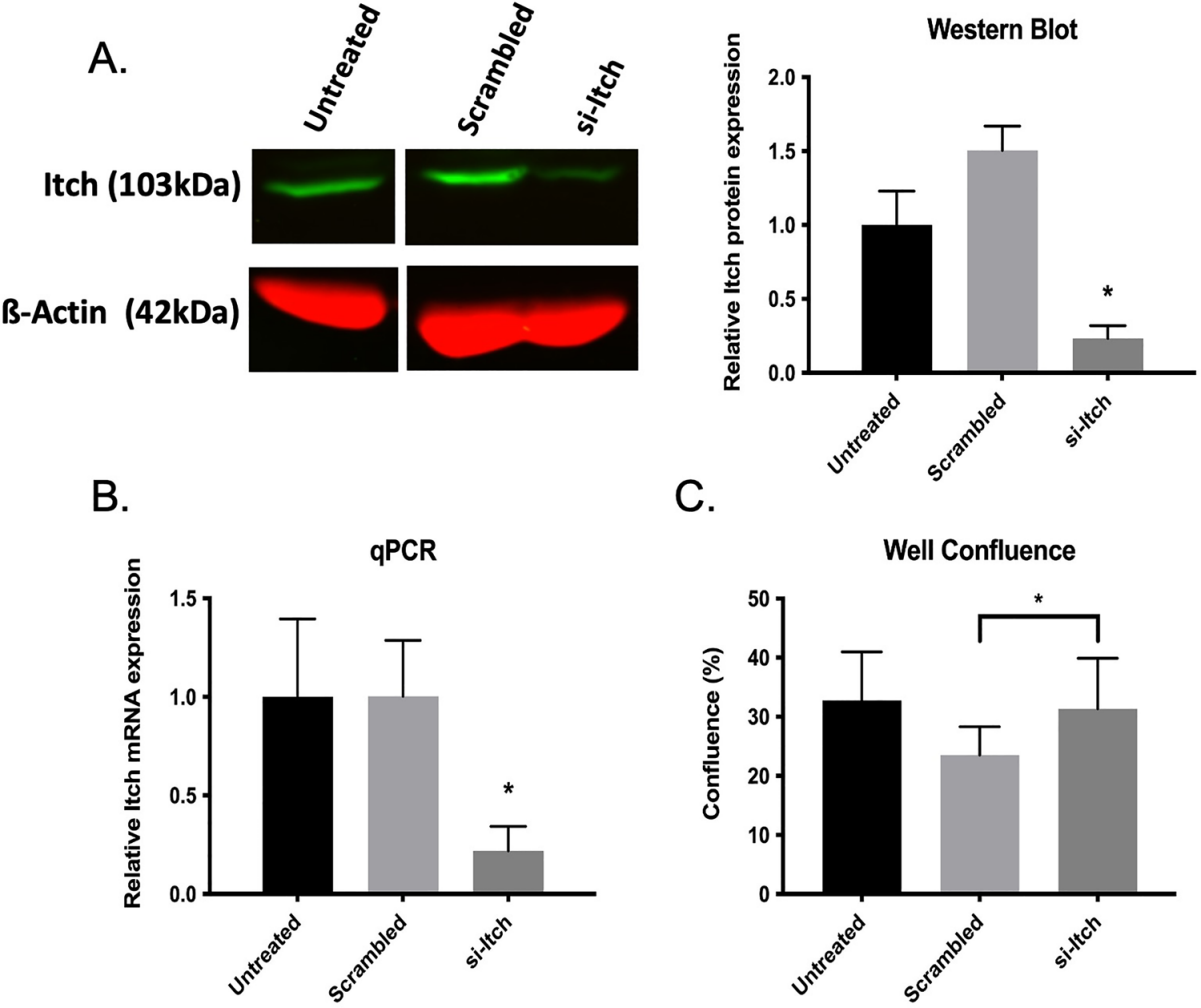
Successful siRNA-mediated knockdown of *Itch* in primary human monocytes: **A** siRNAmediated knockdown of Itch (si-Itch) confirmed by western blot. Itch (green) to β-Actin (red) signal ratio was determined and quantified relative to an untreated control. **B** Itch mRNA quantified by qPCR in untreated, scrambled, and si-Itch treated cells. GAPDH was used as a housekeeping gene. **C** Percentage confluence of wells in a 96-well plate that were either untreated or subject to scrambled or Itch-targeted siRNA. Confluence determined by automated cytometry analysis (n = 20 per condition)

On day 6 cells were exposed to siRNA for 24 h. siRNA-containing media was then replaced with osteoclast differentiation media. Western blot and qPCR were used to compare expression of *Itch* at both the protein level and mRNA level in monocytes. In siRNA-treated samples (si-Itch) there was > 75% reduction in *Itch* protein expression compared to the untreated control and even more so compared to the scrambled control (Fig. 1A). These results were mirrored at the transcription level with an almost 80% reduction in mRNA expression compared to untreated cells (Fig. 1B). This data demonstrates that we can robustly knockdown Itch in primary human monocytes. Little observable difference in β-Actin protein indicated limited or no change in cell viability during knockdown.

After confirmation that we were able to transiently knockdown Itch in primary cells, PBMCs were aliquoted on one of two types of 96-well plates: either plain-surface or Osteo-Surface Assay plates [20] and were cultured for a total of 14 days; 7 days with M-CSF for promoting monocyte attachment and survival and 7 days with RANKL added (differentiation media). Automated cytometry analysis was used to assess well confluence for any differences in cell viability at the end of the culture period (Fig. 1C) to determine if knockdown conditions had any effect. No significant difference was seen for si-Itch-treated samples compared to untreated controls however there was an 8% decrease in confluence for scrambled wells compared to si-Itch (mean = 23% & 31% for scrambled and si-Itch respectively, p = 0.0012). Interestingly in the osteo-surface plate whilst there was no difference in confluence between conditions, overall confluence was on-average 10% lower than that observed in plain-surface 96 well plate (Fig. S1), indicating a reduction in either attachment or viability of cells to the differing plate type.

### siRNA-mediated *Itch* knockdown facilitates mature osteoclast formation

**Fig. 2:**
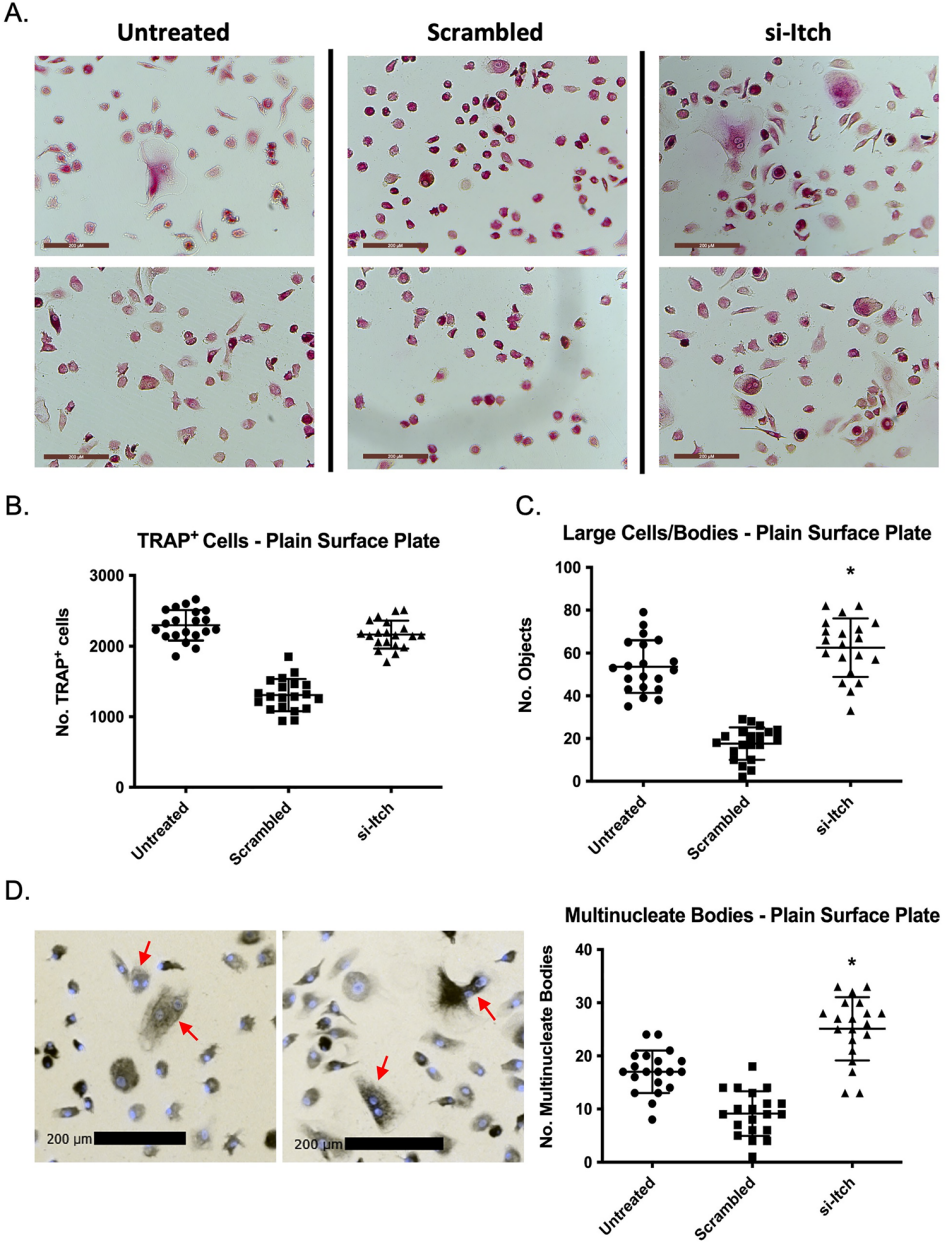
Knockdown of Itch in primary human monocytes increases osteoclast differentiation ex-vivo: **A** TRAP stained cells in wells after siRNA treatment and differentiation period were imaged under a light microscope ( ×10 magnification). Automated cytometry analysis for TRAP + (**B**) and large, TRAP + , cells/bodies (**C**) in wells. **D** Cell nuclei stained with Hoechst 33342 and imaged. Multinucleate bodies (red arrows) were manually counted and results plotted. Box blots show mean and standard deviation within each test condition, statistical significance was determined by Student’s t-test

After 7 days culture in osteoclast differentiation media cells were fixed and stained for TRAP (Fig. 2A), TRAP staining being a classic histological method for identification of osteoclasts [21]. TRAP-positive (TRAP^+^) cells were counted using automated cytometry analysis using a single-cell count algorithm adjusted to identify TRAP^+^ cells in 96-well plates (Fig S2A). Untreated and si-Itch wells had comparable numbers of TRAP^+^ cells (mean = 2294 & 2162 respectively) whilst scrambled wells had less (mean = 1308) (Fig. 2B), concurrent with the confluence data prior to TRAP staining.

Mature, fully differentiated osteoclasts are large multinucleate cells formed from the fusion of several individual premature, mononuclear osteoclasts. Plates were re-analysed with an algorithm designed for TRAP^+^ objects above a surface-area threshold (Fig. S2B). Wells treated with si-Itch had significantly higher numbers of hits compared to untreated cells (Fig. 2C) (mean = 63 & 54 for si-Itch and untreated respectively, p = 0.0376) indicating an increase in the number of mature osteoclasts. Scrambled-treated wells displayed far lower numbers (mean = 18). Data was also mirrored in the osteo-surface assay plate with si-Itch-treated wells having higher numbers of hits (Fig. S2C and D) despite little to no difference in confluence between each condition prior TRAP staining.

To further verify that mature osteoclasts have formed, cells on the plain-surface plate were stained with Hoechst33342 to look for multinucleate bodies. The plate was scanned again and blue fluorescence (461 nm emission) and a brightfield filter and images merged (Fig. 2D). Multinucleate bodies were counted manually (Fig. 2E). *Itch* knockdown resulted in an increase in multinucleate cells (mean = 25) compared to both untreated (mean = 17) and scrambled-treated (mean = 9) wells (t-test comparing si-Itch vs untreated, p < 0.0001). These results combined confirm that genetic knockdown of *Itch* promotes differentiation of PBMCs into cells whose morphology mimics mature osteoclasts, replicating work shown in mice but in a human ex vivo context.

## Discussion

Studies in *Itch-/-* mice have revealed the mechanism by which *Itch* exerts influence upon bone remodelling by inhibiting osteoclast and osteoblast differentiation [9, 10, 22]. Although not all the phenotype of both human *Itch*-mutant cohorts are seen in mouse models, common features suggest a defect in bone growth and remodelling in humans. We examined if genetic knockdown of *Itch* reproduces results seen for mouse osteoclasts in human monocytes ex vivo. *Itch* knockdown increases formation of large, multinucleate TRAP^+^ cells, indicative of increased osteoclast formation.

In adults, upregulation of proteins associated with increased osteoclastogenesis or osteoblastogenesis is usually due to localised inflammation causing persistent NF-κB signalling [9, 22]. In the case of osteoclasts, TRAF6 self-polyubiquitination is required to transduce RANKL/RANK signalling to promote NF-κB transcriptional activation. Mouse studies have identified that the DUB CYLD associates with Itch to deubiquitinate TRAF6 and inhibit NF-κB. Although *Itch* does act to regulate the immune response and inflammatory signalling pathways, the data in this study provides evidence of an intrinsic cellular mechanism by which *Itch* regulates osteoclast differentiation as the conditions were designed to study *Itch* in osteoclasts in a vacuum. During cell culture there was a lack of external inflammatory agents, with only M-CSF and RANKL being added. Furthermore, siRNA acts intracellularly to knockdown gene function through exploitation of endogenous RNA interference machinery.

Although we have shown that siRNA-mediated knockdown in monocytes is robust and repeatable, further optimisation could be performed to broaden the range of applicable gene-editing techniques. *Itch* knockout in monocytes was attempted using CRISPR-Cas9 via lentiviral transduction (Fig. S3). However, it was found that monocytes were refractory to lentiviral transduction, causing significant cell death. As monocytes do not divide, a “pseudo-stable” knockout can in theory be generated from plasmid shRNA strategies.

We observed differences in well confluences between each of the treatment regimens and plate formats. Previous studies have reported that mouse osteoclast culture on dentine discs promoted cell survival [9] although the mechanism is unclear. The osteo-surface assay plate used in our experiments have wells that are coated in a synthetic bone-like matrix. In this study we observed that there was decreased well confluence between the different plate formats however the discrepancy in confluence between the scrambled and other conditions is absent in the osteo-surface assay plate.

A small molecule screen found that the tricyclic antidepressant clomipramine (CMI) can inhibit Itch activity [23]. A later study looked at the effect of CMI treatment on osteoclast formation and bone resorption in WT and *Itch*-/- mice [10] and found increased osteoclast formation in WT mice and increased bone resorption. Meanwhile *Itch*-/- were unaffected by CMI indicating that CMI-dependent bone loss is dependent on interaction with Itch. It would be interesting to see if a treatment of CMI in monocytes produces osteoclast numbers that are comparable to that seen for the siRNA-mediated *Itch* knockdown. There is reported evidence of dose-dependent increase in fracture risk in patients receiving therapeutic CMI [24], however no link was made to Itch regulation and osteoclastogenesis in this cohort.

Our preliminary data shows that siRNA-mediated *Itch* knockdown promotes differentiation of mature osteoclast-like cells from primary human monocytes ex vivo. However, additional experiments need to be performed to characterise the function of these cells to see if they retain the resorptive function of osteoclasts and if over-expression of *Itch* can inhibit differentiation in osteoclast culture conditions. Furthermore, although data from mouse studies elucidates the mechanism by which *Itch* modulates osteoclast differentiation via TRAF6 and CYLD, this still needs to be confirmed for human osteoclasts.

## Supplementary Information

Below is the link to the electronic supplementary material.

Supplementary file1 (PDF 3881 kb)

## Abbreviations

TH2: T-helper type 2 cell
TFH: T-follicular helper cell
T-reg: Regulatory T cell
CMI: Clomipramine
RANK: Receptor activator of nuclear factor-κB
OPG: Osteoprotegrin
RANKL: RANK ligand
TRAF6: TNF receptor-associated factor 6
NF-κB: Nuclear factor-κB
Ub: Ubiquitin
wt: Wild-type
DUB: Deubquitinating enzyme
CYLD: Cylindromatosis
TRAP: Tartrate resistant acid phosphatase
