# Supplementary material for: Silencing Itch in human peripheral blood monocytes promotes their differentiation into osteoclasts
Source: Mol Biol Rep. 2022 Jul 6;49(9):9113–9. doi: 10.1007/s11033-022-07726-1 (PMC9463264; doi:10.1007/s11033-022-07726-1)

Supplementary Figures

Figure S1: A. Percentage confluence of cells in 96-well osteo-surface assay plate after 14 days of culture (n = 20 for each condition). **B.** Comparison of confluence between plain (blue) and osteo-surface (purple) plates for each condition. Student's t-test confirmed statistically significant difference in mean values of untreated and si-Itch wells ($p = 2.33 \times 10^{-5}$ & 2.21×10^{-5} respectively).

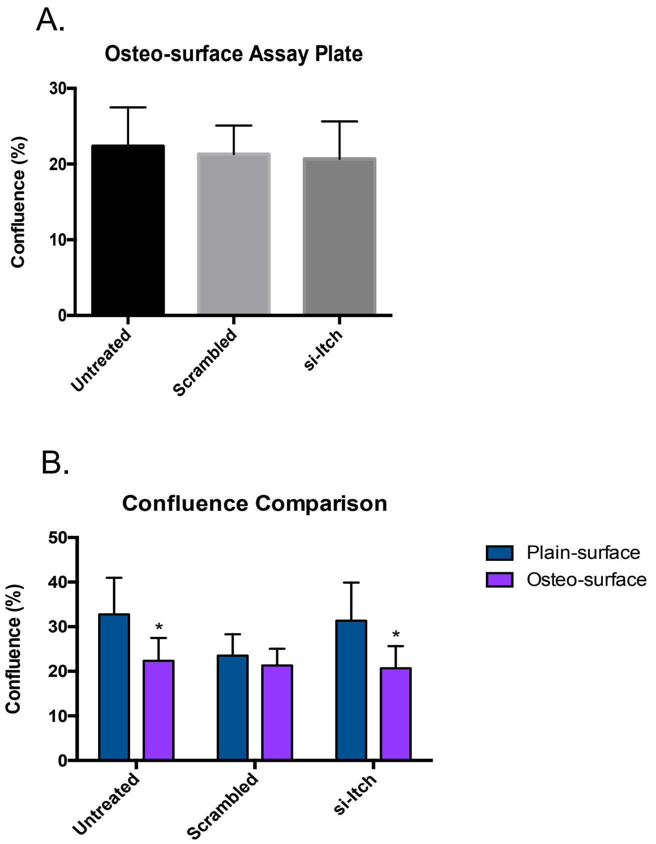

Figure S2: A. Representative images of a section of a well before (left) and after (right) the TRAP⁺ cell counting algorithm was applied. **B.** Representative images of a section of a well before (left) and after (right) the large TRAP⁺ cell/body counting algorithm was applied. Positive hits that fulfil algorithm criteria are highlighted with a green outline. **C.** Quantification of automated cytometry analysis for TRAP⁺ cells in osteo-surface assay plate. Means for untreated, scrambled and si-Itch wells are 449, 330, and 532 respectively. Asterix indicates statistically significant difference between the means of si-Itch and untreated wells ($p = 0.0048$). **D.** Quantification of large, TRAP+, cells/bodies algorithm in wells of osteo-surface assay plate. Means for untreated, scrambled and si-Itch wells are 4, 2, and 9 respectively. Asterix indicates statistically significant difference between the means of si-Itch and untreated wells ($p = 0.009$).

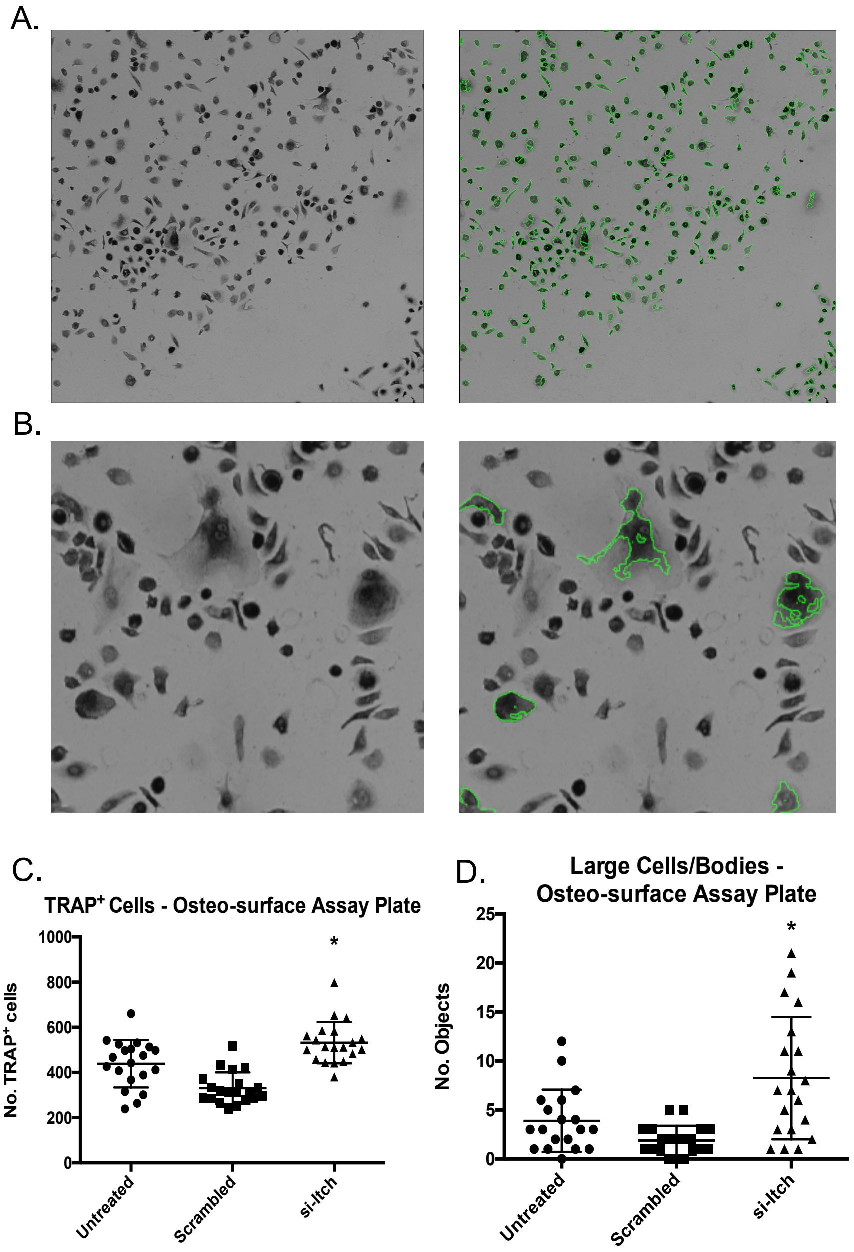

Figure S3: A. Western blot image for Itch and β-Actin used in Figure 1 which includes the lysates for CRISPR-Cas9 treated cells. Cas9 with scrambled gRNA in lane 2 and Cas9 with Itch-targeted gRNA in lane 3. **B.** Whole western blot image.

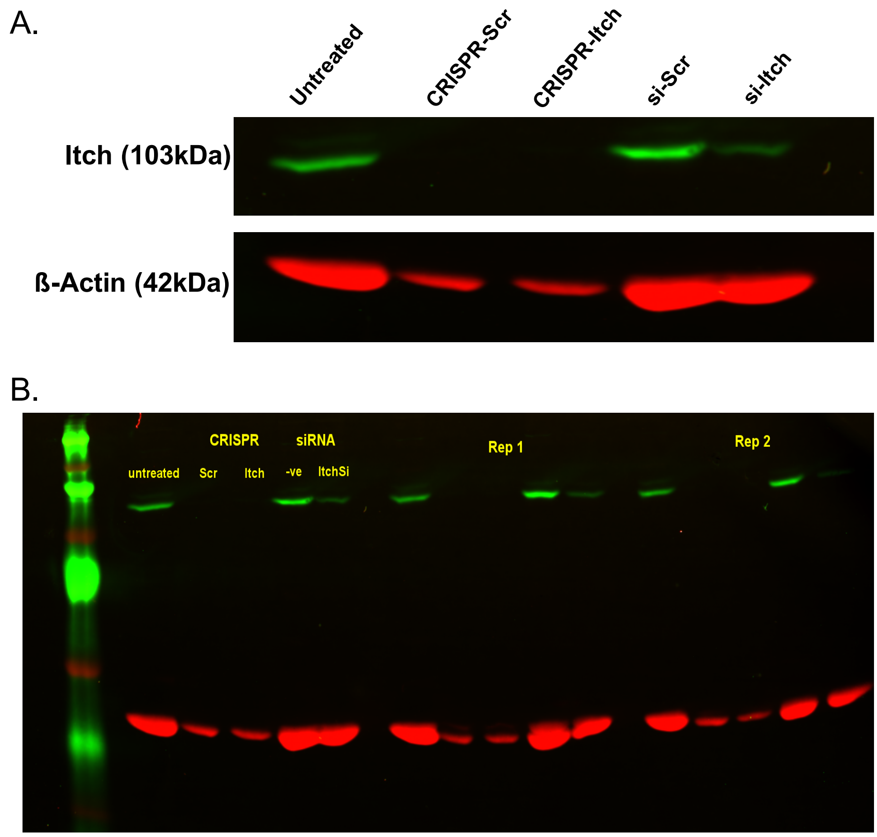

Supplement: Supplementary file 1 — Supplementary file1 (PDF 3881 kb) [file 11033_2022_7726_MOESM1_ESM.pdf]
